# Influence of Functionally Graded Protective Coating on the Temperature in a Braking System

**DOI:** 10.3390/ma16124308

**Published:** 2023-06-10

**Authors:** Aleksander Yevtushenko, Katarzyna Topczewska, Przemysław Zamojski

**Affiliations:** Faculty of Mechanical Engineering, Bialystok University of Technology (BUT), 45C Wiejska Street, 15-351 Bialystok, Poland; a.yevtushenko@pb.edu.pl (A.Y.); p.zamojski@pb.edu.pl (P.Z.)

**Keywords:** braking, frictional heating, functionally graded material, temperature, thermal barrier coating

## Abstract

A mathematical model of heat generation due to friction in a disc–pad braking system was developed with consideration of a thermal barrier coating (TBC) on the friction surface of the disc. The coating was made of functionally graded material (FGM). The three-element geometrical scheme of the system consisted of two homogeneous half-spaces (pad and disc) and a functionally graded coating (FGC) deposited on the friction surface of the disc. It was assumed that the frictional heat generated on the coating-pad contact surface was absorbed to the insides of friction elements along the normal to this surface. Thermal contact of friction between the coating and the pad as well as the heat contact between the coating and the substrate were perfect. On the basis of such assumptions, the thermal friction problem was formulated, and its exact solution was obtained for constant and linearly descending specific friction power over time. For the first case, the asymptotic solutions for small and large values of time were also found. A numerical analysis was performed on an example of the system containing a metal ceramic (FMC-11) pad, sliding on the surface of a FGC (ZrO_2_–Ti-6Al-4V) applied on a cast iron (ChNMKh) disc. It was established that the application of a TBC made of FGM on the surface of a disc could effectively reduce the level of temperature achieved during braking.

## 1. Introduction

Frictional heating occurs when two contacting elements slide against each other, resulting in the conversion of mechanical energy into heat [1,2]. Modern braking systems operate based on this phenomenon [3]. Therefore, the main requirement for friction elements is resistance to elevated temperature, which can be improved by thermal barrier coatings applied on friction surfaces [4,5]. Due to their high thermal and wear resistance, ceramic materials are mostly used in the production of protective coatings for friction components [6,7]. However, in conventional coated elements, cracking may appear on the interface between the layer and a substrate, because of material properties mismatch [8]. To overcome this problem, functionally graded coatings have been introduced that possess smooth gradation of properties to reduce stress concentration on the interface and to reinforce the bond cohesion [9,10].

Accurate models of the frictional heating process are critical in the design of brakes, because they can provide insights into the temperature and thermal stress distributions initiated in the friction elements during braking actions [11]. Such models are developed on the basis of the thermal problems of friction which, in most cases, are obtainable only for bodies bounded by parallel planes (e.g., semi-spaces and strips) [12]. The simplest model used to formulate thermal problems of friction constitutes a single body, obtained after a virtual separation of the friction pair elements. Then, the friction interaction on the contact surface of the elements is replaced by a heat flux with an intensity proportional to the specific friction power. For this purpose, the heat partition ratio is introduced a priori to the model in order to determine the amount of heat absorbed by each element of the friction pair [13]. The most commonly used in the literature is the two-element geometric scheme, which represents both elements of the friction pair and considers heat generation on the contact area due to friction. Another scheme is a three-element model of a tribosystem, consisting of a semi-space sliding on the outer surface of a strip deposited on a semi-infinite substrate. Such a scheme can be adopted to simulate a frictional disc/pad/caliper system, consisting of a coated disc paired with a brake pad. A comparative analysis of the solutions to the thermal problems of friction for a disc brake system, obtained by means of two- and three-element models, was demonstrated in [14]. The three-element scheme considered the influence of the material properties of the caliper, on which the brake pad was placed, on the distribution of temperature and thermal stresses. Other transient thermal problems of friction for braking systems on the basis of a three-element scheme have been studied in [15,16,17,18,19,20,21,22]. The analytical solution to a boundary value problem of heat conduction for such a system was obtained under uniform sliding in a study by [8]. A three-element scheme to formulate thermal problems of friction for single braking with constant deceleration was used in [17,18]. The achieved solutions determined the temperature distribution and corresponding quasi-static thermal stresses in the disc, pad, and caliper of a tribosystem, both in the sliding phase during braking as well as in the cooling phase after braking action. The same scheme was used to consider a three-element braking system, i.e., a top semi-space (the disc) and a strip (the pad) deposited on a substrate (the caliper), in a study by [19]. The heat conduction problem was formulated and solved with time-dependent pressure and the assumption of imperfect thermal contact conditions on the disc–pad interface, in order to study the influence of heat resistance on the thermal behavior of the system. A generalization of this solution for a case considering fluctuations in pressure on the contact surface was presented in [20]. Such a temporal profile of contact pressure during braking with consideration of its oscillations was also considered in [23]. Asymptotic solutions (at large and small values of time) of the heat conduction problem for a three-element tribosystem with generalized boundary conditions on the sliding surface were obtained in [21]. All the mentioned studies concern the thermal problems of friction formulated for homogeneous materials. The thermal problems of friction for a three-element system with a brake pad made of periodic composite material was considered analytically in [22]. The assumption was made that the composite contained four sub-cells with rectangular cross sections, with different thermo-physical properties. The influence of geometrical dimensions of composite sub-cells on the maximum temperature in the system was investigated. However, modern friction elements can have far more complicated internal structure, such as functionally graded materials (FGMs), which have continuously changing properties throughout the volume of material [24]. Some analytical solutions to the heat conduction problems formulated for braking systems with functionally graded friction materials have already been obtained in [25,26,27,28,29,30]. However, these studies concerned simpler geometrical schemes, including a two FGM semi-spaces system [25,26]; functionally graded semi-infinite body coupled with homogeneous element [27,28]; and a one-element system consisting of a heating semi-space with deposited functionally graded coating, heated by the frictional heat flux on the friction surface [29]. A comparative analysis of frictional heating models formulated for FGMs by means of two-element and one-element schemes was performed in [30]. Based on the obtained results, a new heat partition ratio formula was proposed for a functionally graded friction couple.

In our previous paper [29], the problem we considered was a coated brake disc simulated using a homogeneous substrate with a deposited coating made of functionally graded material. The outer surface of the coating was assumed to be heated by the frictional heat flux. The current study is a continuation of that study, by introducing a second element in a friction pair, i.e., a brake pad, as the counterbody in a tribosystem. In this study, the thermal problem of friction is considered for a three-element system consisting of two homogeneous semi-spaces (pad and disc) and a functionally graded coating (FGC) is deposited on the friction surface of the disc, considering the heat generation on the disc–pad interface due to the friction during braking. The mathematical model obtained was based on the following algorithm:

(1) Formulation of the proper boundary value problem of heat conduction.
(2) Transition of the problem to the Laplace integral space.
(3) Finding the solutions in the form of Laplace transforms.
(4) Proceeding from the transforms of the solutions to the originals.
(5) Verification of the obtained solutions.
(6) Designating the asymptotic solutions for small and high values of the Fourier number.
(7) Performance of a numerical analysis for a selected case.

## 2. Problem Formulation

**Figure 1:**
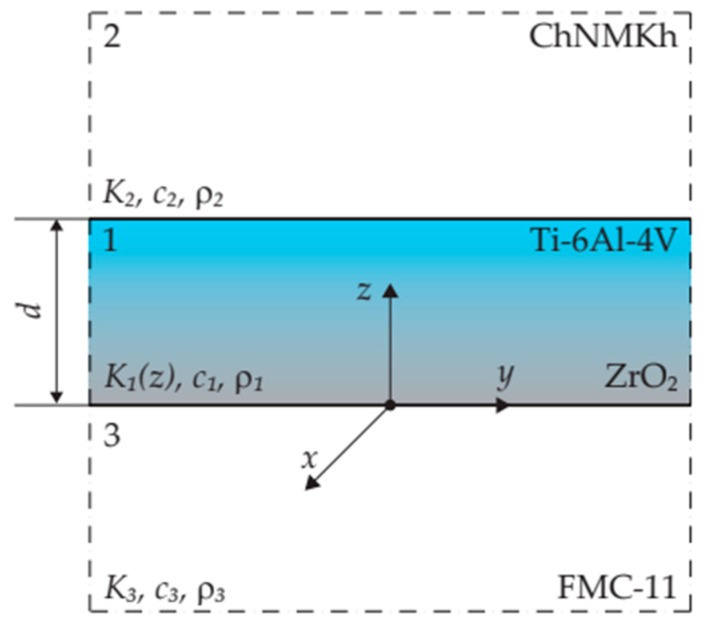
Scheme of the considered three-element tribosystem.

In this study, we consider the sliding contact of two semi-infinite bodies (half-spaces) taking into consideration the generation of heat due to friction (Figure 1). The upper half-space consists of a protective coating applied on the surface of the substrate. The materials of the substrate and lower semi-space (counterbody) are homogeneous, so their properties, i.e., thermal conductivity ($K_{l}$), specific heat ($c_{l}$), and density ($\rho_{l}$) are uniform throughout volumes of the elements, whereas the coating is made of a two-element functionally gradient material (FGM) with a thermal conductivity coefficient $K_{1}$ increasing exponentially along its thickness [31]:

(1) $$K_{1}(z)=K_{1,1}e^{\gamma^{*}z/d},\;\gamma^{*}=\ln(K_{1,2}\;K_{1,1}^{-1}),\;0\leq z\leq d,$$

where d is the thickness of the coating, $\gamma^{*}\geq 0$ is the gradient parameter FGM [32], $K_{1,1}\equiv K_{1}(0)$ and $K_{1,2}\equiv K_{1}(d)$ are the thermal conductivity coefficients of the FGM constituents, and z is the spatial coordinate in the axial direction.

At the initial moment t=0, the temperature T of all bodies in the system are constant and equal to $T_{0}$. Next, both semi-spaces, under the effect of pressure $p_{0}$ and acting parallel to the axis Oz, come into contact and simultaneously begin to slide in the positive direction of the axis Ox with constant velocity $V_{0}$. Due to friction on the contact surface z=0, heat is generated, and the bodies heat up. Assuming that the friction thermal contact is perfect, i.e., at a set moment of time t>0 the friction surfaces of the coating and counterbody are heated to the same temperature, and the sum of the intensities of heat fluxes, directed to the insides of the coating and counterbody along the normal to the contact surface, is equal to the specific friction power $q_{0}=fp_{0}V_{0}$, where f is the friction coefficient. The thermal connection of the coating with the substrate is perfect, i.e., the temperature and intensity of heat fluxes of these elements at the interface z=d are the same. Thermal sensitivity of materials and wear are neglected. All assumptions in more detail are listed in our previous papers [25,26]. The aim of this study is to develop a mathematical model for the analytical determination of the temperature T of the considered three-element system at a fixed location $\left|z\right|<\infty$ at the selected time moment t>0:

(2) $$\frac{\partial }{\partial z}\left[K_{1}(z)\frac{\partial \Theta (z,t)}{\partial z}\right]=\rho_{1}c_{1}\frac{\partial \Theta (z,t)}{\partial t},\;0<z<d,\;t>0,$$

(3) $$K_{2}\frac{\partial^{2}\Theta (z,t)}{\partial z^{2}}=\rho_{2}c_{2}\frac{\partial \Theta (z,t)}{\partial t},\;z>d,\;t>0,$$

(4) $$K_{3}\frac{\partial^{2}\Theta (z,t)}{\partial z^{2}}=\rho_{3}c_{3}\frac{\partial \Theta (z,t)}{\partial t},\;z<d,\;t>0,$$

(5) $$\Theta (0^{+},t)=\Theta (0^{-},t),\;t>0,$$

(6) $${\left.K_{1}(z)\frac{\partial \Theta (z,t)}{\partial z}\right|}_{z=0^{+}}-{\left.K_{3}\frac{\partial \Theta (z,t)}{\partial z}\right|}_{z=0^{-}}=-q_{0},\;t>0,$$

(7) $$\Theta (d^{+},t)=\Theta (d^{-},t),\;t>0,$$

(8) $${\left.K_{1}(z)\frac{\partial \Theta (z,t)}{\partial z}\right|}_{z=d^{+}}={\left.K_{2}\frac{\partial \Theta (z,t)}{\partial z}\right|}_{z=d^{-}},\;t>0,$$

(9) $$\Theta (z,t)\to 0,\;\left|z\right|\to \infty ,\;t>0,$$

(10) $$\Theta (z,0)=0,\;\left|z\right|<\infty ,$$

where $K_{2}$ and $K_{3}$ are the thermal conductivities of the substrate and counterbody materials, respectively; $\rho_{l}$ and $c_{l}$, respectively, are the density and specific heat of materials of the coating (l=1), the substrate (l=2), and the counterbody (l=3).

Incorporating the following dimensionless variables and parameters:

(11) $$\zeta =\frac{z}{d},\;\tau =\frac{k_{1}t}{d^{2}},\;K_{3}^{*}=\frac{K_{3}}{K_{1,1}},\;k_{2}^{*}=\frac{k_{2}}{k_{1}},\;k_{3}^{*}=\frac{k_{2}}{k_{1}},\;\Theta^{*}=\frac{\Theta }{\Lambda },$$

where

(12) $$k_{1}=\frac{K_{1,1}}{c_{1}\rho_{1}},\;k_{2}=\frac{K_{2}}{c_{2}\rho_{2}},\;k_{3}=\frac{K_{3}}{c_{3}\rho_{3}},\;\Lambda =\frac{q_{0}d}{K_{1,1}},$$

Problem (2)–(10) was written in the form:

(13) $$\frac{\partial^{2}\Theta^{*}(\zeta ,\tau )}{\partial \zeta^{2}}+\gamma^{*}\frac{\partial \Theta^{*}(\zeta ,\tau )}{\partial \zeta }-e^{-\gamma^{*}\zeta }\frac{\partial \Theta^{*}(\zeta ,\tau )}{\partial \tau }=0,\;0<\zeta <1,\;\tau >0,$$

(14) $$\frac{\partial^{2}\Theta^{*}(\zeta ,\tau )}{\partial \zeta^{2}}-\frac{1}{k_{2}^{*}}\frac{\partial \Theta^{*}(\zeta ,\tau )}{\partial \tau }=0,\;\zeta >1,\;\tau >0,$$

(15) $$\frac{\partial^{2}\Theta^{*}(\zeta ,\tau )}{\partial \zeta^{2}}-\frac{1}{k_{3}^{*}}\frac{\partial \Theta^{*}(\zeta ,\tau )}{\partial \tau }=0,\;\zeta <0,\;\tau >0,$$

(16) $$\Theta^{*}(0^{+},\tau )=\Theta^{*}(0^{-},\tau ),\;\tau >0,$$

(17) $${\left.\frac{\partial \Theta^{*}(\zeta ,\tau )}{\partial \zeta }\right|}_{\zeta =0^{+}}-{\left.K_{3}^{*}\frac{\partial \Theta^{*}(\zeta ,\tau )}{\partial \zeta }\right|}_{\zeta =0^{-}}=-1,\;\tau >0,$$

(18) $$\Theta (1^{+},\tau )=\Theta (1^{-},\tau ),\;\tau >0,$$

(19) $${\left.e^{\gamma^{*}}\frac{\partial \Theta^{*}(\zeta ,\tau )}{\partial \zeta }\right|}_{\zeta =1^{+}}={\left.K_{2}^{*}\frac{\partial \Theta^{*}(\zeta ,\tau )}{\partial \zeta }\right|}_{\zeta =1^{-}},\;\tau >0,$$

(20) $$\Theta^{*}(\zeta ,\tau )\to 0,\;\left|\zeta \right|\to \infty ,\;\tau >0,$$

(21) $$\Theta^{*}(\zeta ,0)=0,\;\left|\zeta \right|<\infty .$$

## 3. Exact Solution

By means of the Laplace integral transform [33]:

(22) Θ¯*(ζ,p)≡L[Θ*(ζ,τ);p]=∫0∞Θ*(ζ,τ)e−pτdτ, Rep≥0,

the boundary value problem (13)–(21) was reduced to the following boundary problem for a system of three ordinary differential equations of the second order:

(23) $$\frac{d^{2}{\bar{\Theta }}^{*}(\zeta ,p)}{d\zeta^{2}}+\gamma^{*}\frac{d{\bar{\Theta }}^{*}(\zeta ,\tau )}{d\zeta }-pe^{-\gamma^{*}\zeta }{\bar{\Theta }}^{*}(\zeta ,p)=0,\;0<\zeta <1,$$

(24) $$\frac{d^{2}{\bar{\Theta }}^{*}(\zeta ,p)}{d\zeta^{2}}-\frac{p}{k_{2}^{*}}{\bar{\Theta }}^{*}(\zeta ,p)=0,\;\zeta >1,$$

(25) $$\frac{d^{2}{\bar{\Theta }}^{*}(\zeta ,p)}{d\zeta^{2}}-\frac{p}{k_{3}^{*}}{\bar{\Theta }}^{*}(\zeta ,p)=0,\;\zeta <0,$$

(26) $${\bar{\Theta }}^{*}(0^{+},p)={\bar{\Theta }}^{*}(0^{-},p),$$

(27) $${\left.\frac{d{\bar{\Theta }}^{*}(\zeta ,p)}{d\zeta }\right|}_{\zeta =0^{+}}-{\left.K_{3}^{*}\frac{d{\bar{\Theta }}^{*}(\zeta ,p)}{d\zeta }\right|}_{\zeta =0^{-}}=-\frac{1}{p},$$

(28) $${\bar{\Theta }}^{*}(1^{+},p)={\bar{\Theta }}^{*}(1^{-},p),$$

(29) $${\left.e^{\gamma^{*}}\frac{d{\bar{\Theta }}^{*}(\zeta ,p)}{d\zeta }\right|}_{\zeta =1^{+}}={\left.K_{2}^{*}\frac{d{\bar{\Theta }}^{*}(\zeta ,p)}{d\zeta }\right|}_{\zeta =1^{-}},$$

(30) $${\bar{\Theta }}^{*}(\zeta ,p)\to 0,\;\left|\zeta \right|\to \infty .$$

The solution to problem (23)–(30) has the following form:

(31) $${\bar{\Theta }}^{*}(\zeta ,p)=e^{-\tilde{\alpha }\zeta }\;{\bar{\Theta }}_{0}^{*}(p){\bar{\Theta }}_{1}^{*}(\zeta ,p),\;0\leq \zeta \leq 1,$$

(32) $${\bar{\Theta }}^{*}(\zeta ,p)=\tilde{\alpha }\;{\bar{\Theta }}_{0}^{*}(p){\bar{\Theta }}_{2}^{*}(\zeta ,p),\;\zeta \geq 1,$$

(33) $${\bar{\Theta }}^{*}(\zeta ,p)={\bar{\Theta }}_{0}^{*}(p){\bar{\Theta }}_{3}^{*}(\zeta ,p),\;\zeta <0,$$

where

(34) $${\bar{\Theta }}_{0}^{*}(p)=\frac{1}{\sqrt{p}},\;{\bar{\Theta }}_{1}^{*}(\zeta ,p)=\frac{\Delta_{1}(\zeta ,p)}{p\Delta (p)},\;{\bar{\Theta }}_{2}^{*}(\zeta ,p)=\frac{e^{-\zeta_{2}\sqrt{p}}}{p\sqrt{p}\Delta (p)},\;{\bar{\Theta }}_{3}^{*}(\zeta ,p)=\frac{\Delta_{3}(p)e^{-\zeta_{3}\sqrt{p}}}{p\Delta (p)},$$

(35) $$\Delta_{1}(\zeta ,p)=A(p{)I}_{1}(\zeta_{1}\sqrt{p})+Β(p{)K}_{1}(\zeta_{1}\sqrt{p}),\;\Delta_{3}(p)=A(p{)I}_{1}(\alpha \sqrt{p})+Β(p{)K}_{1}(\alpha \sqrt{p}),$$

(36) $$\Delta (p)=A(p{)[I}_{0}(\alpha \sqrt{p})+\varepsilon_{3}I_{1}(\alpha \sqrt{p})]-B(p{)[K}_{0}(\alpha \sqrt{p})-\varepsilon_{3}K_{1}(\alpha \sqrt{p})],$$

(37) $$A(p)=K_{0}(\beta \sqrt{p})+\varepsilon_{2}\;e^{-\tilde{\alpha }}K_{1}(\beta \sqrt{p}),\;B(p)=I_{0}(\beta \sqrt{p})-\varepsilon_{2}\;e^{-\tilde{\alpha }}I_{1}(\beta \sqrt{p}),$$

(38) $$\alpha =\frac{2}{\gamma^{*}},\tilde{\alpha }=\frac{1}{\alpha },\beta =\frac{\alpha }{e^{\tilde{\alpha }}},\varepsilon_{2}=\frac{K_{2}^{*}}{\sqrt{k_{2}^{*}}},\;\varepsilon_{3}=\frac{K_{3}^{*}}{\sqrt{k_{3}^{*}}},\;\zeta_{1}=\frac{\alpha }{e^{\tilde{\alpha }\zeta }},\;\zeta_{2}=\frac{\zeta -1}{\sqrt{k_{2}^{*}}},\zeta_{3}=\frac{\left|\zeta \right|}{\sqrt{k_{3}^{*}}},$$

where $I_{n}(x)$ and $K_{n}(x)$ are the modified Bessel functions of the nth order of the first and second kind, respectively [34].

Considering the forms of the transformed solutions (31)–(33) and based on the convolution theorem of the two functions, the dimensionless temperature rises were found [33]:

(39) $$\Theta^{*}(\zeta ,\tau )=e^{-\tilde{\alpha }\zeta }\int_{0}^{\tau }\Theta_{0}^{*}(\tau -s)\Theta_{1}^{*}(\zeta ,s)ds,\;0\leq \zeta \leq 1,\;\tau \geq 0,$$

(40) $$\Theta^{*}(\zeta ,\tau )=\tilde{\alpha }\int_{0}^{\tau }\Theta_{0}^{*}(\tau -s)\Theta_{2}^{*}(\zeta ,s)ds,\;\zeta \geq 1,\;\tau \geq 0,$$

(41) $$\Theta^{*}(\zeta ,\tau )=\int_{0}^{\tau }\Theta_{0}^{*}(\tau -s)\Theta_{3}^{*}(\zeta ,s)ds,\;\zeta \leq 0,\;\tau \geq 0,$$

where

(42) $$\Theta_{0}^{*}(\tau )\equiv L^{-1}[{\bar{\Theta }}_{0}^{*}(p);\tau ]=\frac{1}{\sqrt{\pi \tau }},$$

(43) Θl∗(ζ,τ)≡L−1[Θ¯l∗(ζ,p); τ]=12πi∫ω−i ∞ω+i ∞Θ¯l∗(ζ,p)epτdp, l=1,2,3, ω≡Rep>0, i≡−1.

Integration on the complex plane (Rep, Imp) in Equation (43) was carried out according to the methodology described in detail in [29] and using the following relations [34]:

(44) $$I_{0}(\pm ix)=J_{0}(x),\;K_{0}(\pm ix)=-0.5\pi [Y_{0}(x)\pm iJ_{0}\left(x\right)],$$

(45) $$I_{1}(\pm ix)=\pm iJ_{1}(ix),\;K_{1}(\pm ix)=-0.5\pi [J_{1}(x)\mp iY_{1}\left(x\right)],$$

where $J_{n}(x)$ and $Y_{n}(x)$ are Bessel functions of the nth order of the first and second kind, respectively. As a result, it was obtained:

(46) $$\Theta_{1}^{*}(\zeta ,\tau )=\frac{e^{\tilde{\alpha }\zeta }}{\varepsilon_{2}+\varepsilon_{3}}+\frac{2}{\pi }\int_{0}^{\infty }\frac{\Phi_{1}(\zeta ,x)}{x\Psi (x)}e^{-x^{2}\tau }dx,\;0\leq \zeta \leq 1,\;\tau \geq 0,$$

(47) $$\Theta_{2}^{*}(\zeta ,\tau )=\frac{\alpha }{\varepsilon_{2}+\varepsilon_{3}}-\frac{4}{\pi^{2}}\int_{0}^{\infty }\frac{\Phi_{2}(\zeta ,x)}{x^{2}\Psi (x)}e^{-x^{2}\tau }dx,\;\zeta \geq 1,\;\tau \geq 0,$$

(48) $$\Theta_{3}^{*}(\zeta ,\tau )=\frac{1}{\varepsilon_{2}+\varepsilon_{3}}+\frac{2}{\pi }\int_{0}^{\infty }\frac{\Phi_{3}(\zeta ,x)}{x\Psi (x)}e^{-x^{2}\tau }dx,\;\zeta \leq 0,\;\tau \geq 0,$$

where

(49) $$\Phi_{1}(\zeta ,x)=\Delta_{R}(x)\Delta_{1,I}(\zeta_{1},x)-\Delta_{I}(x)\Delta_{1,R}(\zeta_{1},x),$$

(50) $$\Phi_{2}(\zeta ,x)=\Delta_{R}(x)\cos(\zeta_{2}x)-\Delta_{I}(x)\sin(\zeta_{2}\;x),$$

(51) $$\begin{array}{l} \Phi_{3}(\zeta ,x)=[\Delta_{R}(x)\Delta_{3,I}(x)-\Delta_{I}(x)\Delta_{3,R}(x)]\cos(\zeta_{3}x)-\\ \;\;\;\;\;\;\;\;\;\;\;\;\;\;\;\;\;\;\;\;\;\;\;\;\;\;\;\;\;\;\;\;\;\;\;\;[\Delta_{R}(x)\Delta_{3,R}(x)+\Delta_{I}(x)\Delta_{3,I}(x)]\sin(\zeta_{3}x)\;, \end{array}$$

(52) $$\Psi (x)=\Delta_{R}^{2}(x)+\Delta_{I}^{2}(x),$$

(53) $$\Delta_{R}(x)=Y_{0}(\alpha x)J_{0}(\beta x)-J_{0}(\alpha x)Y_{0}(\beta x)+\varepsilon_{3}\Delta_{3,R}(x),$$

(54) $$\Delta_{I}(x)=\varepsilon_{3}\Delta_{3,I}(x)-\varepsilon_{2}e^{-\tilde{\alpha }}[Y_{0}(\alpha x)J_{1}(\beta x)-J_{0}(\alpha x)Y_{1}(\beta x)],$$

(55) $$\Delta_{1,R}(\zeta ,x)=\varepsilon_{2}e^{-\tilde{\alpha }}{[J}_{1}(\beta x)Y_{1}(\zeta_{1}x)-Y_{1}(\beta x)J_{1}(\zeta_{1}x)],$$

(56) $$\Delta_{1,I}(\zeta ,x)=J_{0}(\beta x)Y_{1}(\zeta_{1}x)-Y_{0}(\beta x)J_{1}(\zeta_{1}x),$$

(57) $$\Delta_{3,R}(x)=\varepsilon_{2}e^{-\tilde{\alpha }}{[Y}_{1}(\alpha x)J_{1}(\beta x)-J_{1}(\alpha x)Y_{1}(\beta x)],$$

(58) $$\Delta_{3,I}(x)=Y_{1}(\alpha x)J_{0}(\beta x)-J_{1}(\alpha x)Y_{0}(\beta x).$$

Taking into consideration the functions $\Theta_{0}^{*}(\tau )$ (42) and $\Theta_{l}^{*}(\zeta ,\tau )$, l=1, 2, 3 (46)–(48) in Equations (39)–(41), after performing the integration, the sought dimensionless temperature rise was found in the form:

(59) $$\Theta^{*}(\zeta ,\tau )=2\sqrt{\frac{\tau }{\pi }}\left[\frac{1}{\varepsilon_{2}+\varepsilon_{3}}+\frac{2}{\pi }e^{-0.5\gamma^{*}\zeta }\int_{0}^{\infty }\frac{\Phi_{1}(\zeta ,x)}{x\Psi (x)}F(x\sqrt{\tau })dx\right],\;0\leq \zeta \leq 1,\;\tau \geq 0,$$

(60) $$\Theta^{*}(\zeta ,\tau )=2\sqrt{\frac{\tau }{\pi }}\left[\frac{1}{\varepsilon_{2}+\varepsilon_{3}}-\frac{2\gamma^{*}}{\pi^{2}}\int_{0}^{\infty }\frac{\Phi_{2}(\zeta ,x)}{x^{2}\Psi (x)}F(x\sqrt{\tau })dx\right],\;\zeta \geq 1,\;\tau \geq 0,$$

(61) $$\Theta^{*}(\zeta ,\tau )=2\sqrt{\frac{\tau }{\pi }}\left[\frac{1}{\varepsilon_{2}+\varepsilon_{3}}+\frac{2}{\pi }\int_{0}^{\infty }\frac{\Phi_{3}(\zeta ,x)}{x\Psi (x)}F(x\sqrt{\tau })dx\right],\;\zeta \leq 0,\;\tau \geq 0,$$

where

(62) $$F(x)=\frac{e^{-x^{2}}}{x}\int_{0}^{x}e^{s^{2}}ds.$$

To calculate the function F(x) (62) the following approximation formulas were used [35]:

(63) $$F(x)=\sum_{n=0}^{\infty }{\left(-1\right)}^{n}\frac{{\left(2x^{2}\right)}^{n}}{(2n+1)!!},\;0<x<3,\;F(x)=\sum_{n=0}^{N}\frac{(2n-1)!!}{{\left(2x^{2}\right)}^{n+1}},\;x\geq 3.$$

where (−1)!!=1, (2n+1)!!=1×3×5×…×(2n+1).

## 4. Verification of the Solution

Correctness of solutions (59)–(61) will be shown by proving that they satisfy the boundary conditions (16)–(20) and the initial condition (21). By comparing the forms of solutions (59) and (61), it follows that the Equation condition (16) of the temperature of the coating and counterbody on the contact surface ζ=0 will be met if:

(64) $$\Phi_{1}(0^{+},x)=\Phi_{3}(0^{-},x).$$

Substituting in Formulas (55)–(58) ζ=0 ($\zeta_{1}=\alpha$) it was found that

(65) $$\Delta_{1,R}(0,x)=\Delta_{3,R}(x),\;\Delta_{1,I}(0,x)=\Delta_{3,I}(x),$$

from where, on the basis of Equations (49) and (51), we obtain the Equation (64).

Comparing solutions (59) and (60), it can be seen that condition (18) of the temperature equality at the interface ζ=1 will be met, when

(66) $$e^{-0.5\gamma^{*}}\pi x\;\Phi_{1}(1^{+},x)+\gamma^{*}\Phi_{2}(1^{-},x)=0.$$

For ζ=1 from Equation (38), it follows that $\zeta_{1}=\beta$ and $\zeta_{2}=0$. Then, Formulas (55) and (56) yield [34]:

(67) $$\Delta_{1,R}(1,x)=0,\;\Delta_{1,I}(1,x)=J_{0}(\beta x)Y_{1}(\beta \;x)-Y_{0}(\beta x)J_{1}(\beta \;x)\equiv -2{\left(\pi \beta x\right)}^{-1}.$$

Including definition (38) of parameter β, from Equations (49), (50) and (67) it was found:

(68) $$\Phi_{1}(1^{+},x)=-\frac{\gamma^{*}e^{0.5\gamma^{*}}}{\pi x}\Delta_{R}(x),\;\Phi_{2}(1^{-},x)=\Delta_{R}(x),$$

which proves that Equation (66) is fulfilled, which means that the boundary condition (18) is met.

After differentiating solution (59) with respect to a spatial variable, ζ (the corresponding derivatives hereinafter are denoted by the symbol « ’ ») was obtained:

(69) $${\Theta^{\prime }}^{*}(\zeta ,\tau )=\frac{2}{\pi }e^{-0.5\gamma^{*}\zeta }\sqrt{\tau }\;\int_{0}^{\infty }\frac{\left[\Phi_{1}^{\prime }(\zeta ,x)-0.5\gamma^{*}\Phi_{1}(\zeta ,x)\right]}{x\Psi (x)}F(x\sqrt{\tau })dx,\;0\leq \zeta \leq 1,\;\tau \geq 0,$$

where, from Equation (49), it yields:

(70) $$\Phi_{1}^{\prime }(\zeta ,x)=\Delta_{R}(x)\Delta_{1,I}^{\prime }(\zeta ,x)-\Delta_{I}(x)\Delta_{1,R}^{\prime }(\zeta ,x).$$

Considering derivatives [34]:

(71) $$J_{1}^{\prime }(x)=J_{0}(x)-x^{-1}J_{1}(x),\;Y_{1}^{\prime }(x)=Y_{0}(x)-x^{-1}Y_{1}(x),$$

from Formulas (53)–(56) it was found:

(72) $$\Delta_{1,R}^{\prime }(\zeta ,x)=0.5\gamma^{*}\Delta_{1,R}(\zeta ,x)-xe^{-0.5\gamma^{*}\zeta }{\hat{\Delta }}_{I}(\zeta ,x),$$

(73) $$\Delta_{1,I}^{\prime }(\zeta ,x)=0.5\gamma^{*}\Delta_{1,I}(\zeta ,x)-xe^{-0.5\gamma^{*}\zeta }{\hat{\Delta }}_{R}(\zeta ,x),$$

where

(74) $${\hat{\Delta }}_{R}(\zeta ,x)=J_{0}(\beta x)Y_{0}(\zeta_{1}x)-Y_{0}(\beta x)J_{0}(\zeta_{1}x),$$

(75) $${\hat{\Delta }}_{I}(\zeta ,x)=\varepsilon_{2}e^{-0.5\gamma^{*}}{[J}_{1}(\beta x)Y_{0}(\zeta_{1}x)-Y_{1}(\beta x)J_{0}(\zeta_{1}x)].$$

Substituting derivatives (72)–(75) to the right side of Equation (70), it was obtained:

(76) $$\Phi_{1}^{\prime }(\zeta ,x)=0.5\gamma^{*}\Phi_{1}(\zeta ,x)-xe^{-0.5\gamma^{*}\zeta }{\hat{\Phi }}_{1}(\zeta ,x),$$

where

(77) $${\hat{\Phi }}_{1}(\zeta ,x)=\Delta_{R}(x){\hat{\Delta }}_{R}(\zeta ,x)-\Delta_{I}(x){\hat{\Delta }}_{I}(\zeta ,x).$$

Taking into consideration the Formulas (76) and (77), the derivative of function $\Phi_{1}(\zeta ,x)$ in Equation (69) gives:

(78) $${\Theta^{\prime }}^{*}(\zeta ,\tau )=-\frac{2}{\pi }e^{-\gamma^{*}\zeta }\sqrt{\tau }\;\int_{0}^{\infty }\frac{{\hat{\Phi }}_{1}(\zeta ,x)}{\Psi (x)}\;F(x\sqrt{\tau })dx,\;0\leq \zeta \leq 1,\;\tau \geq 0.$$

Next, differentiating solutions (60) and (61), it was found:

(79) $${\Theta^{\prime }}^{*}(\zeta ,\tau )=-\frac{2\gamma^{*}}{\pi^{2}}\sqrt{\tau }\;\int_{0}^{\infty }\frac{\Phi_{2}^{\prime }(\zeta ,x)}{x^{2}\Psi (x)}\;F(x\sqrt{\tau })dx,\;\zeta \geq 1,\;\tau \geq 0,$$

(80) $${\Theta^{\prime }}^{*}(\zeta ,\tau )=\frac{2}{\pi }\sqrt{\tau }\;\int_{0}^{\infty }\frac{\Phi_{3}^{\prime }(\zeta ,x)}{x\Psi (x)}\;F(x\sqrt{\tau })dx,\;\zeta \leq 0,\;\tau \geq 0,$$

where, based on relations (50)–(54) and (57) and (58), the following was determined:

(81) $$\Phi_{2}^{\prime }(\zeta ,x)=-\frac{x}{\sqrt{k_{2}^{*}}}[\Delta_{R}(x)\sin(\zeta_{2}x)+\Delta_{I}(x)\cos(\zeta_{2}\;x)],$$

(82) $$\begin{array}{l} \Phi_{3}^{\prime }(\zeta ,x)=-\frac{x}{\sqrt{k_{3}^{*}}}\{[\Delta_{R}(x)\Delta_{3,I}(x)-\Delta_{I}(x)\Delta_{3,R}(x)]\sin(\zeta_{3}x)-\\ \;\;\;\;\;\;\;\;\;\;\;\;\;\;\;\;\;\;\;\;\;\;\;\;\;\;\;\;\;\;\;\;[\Delta_{R}(x)\Delta_{3,R}(x)+\Delta_{I}(x)\Delta_{3,I}(x)]\cos(\zeta_{3}x)\}. \end{array}$$

Substituting the derivatives (78), (80), and (82) for ζ=0 ($\zeta_{1}=\alpha$,$\zeta_{3}=0$) to the left side of boundary condition (17), it was achieved:

(83) $${\Theta^{\prime }}^{*}(0^{+},\tau )-K_{3}^{*}{\Theta^{\prime }}^{*}(0^{-},\tau )=-\frac{2}{\pi }\sqrt{\tau }\;\int_{0}^{\infty }\frac{\left\{{\hat{\Phi }}_{1}(0,x)+\varepsilon_{3}[\Delta_{R}(x)\Delta_{3,R}(x)+\Delta_{I}(x)\Delta_{3,I}(x)]\right\}}{\Psi (x)}\;F(x\sqrt{\tau })dx,\;\tau \geq 0.$$

Considering relation (77), the following was written:

(84) $$\begin{array}{l} {\hat{\Phi }}_{1}(0,x)+\varepsilon_{3}[\Delta_{R}(x)\Delta_{3,R}(x)+\Delta_{I}(x)\Delta_{3,I}(x)]=\\ \;\;\;\;\;\;\;\;\;\;\;\;\;\;\;\;\;\;\;\;\;\;\;\;\;\;\;\;\;\;\;\;\;\;\;\;\;\;\;\;\;\;\;\;\;\;\;\;\;\Delta_{R}(x)[{\hat{\Delta }}_{R}(0,x)+\varepsilon_{3}\Delta_{3,R}(x)]-\Delta_{I}(x)[{\hat{\Delta }}_{I}(0,x)-\varepsilon_{3}\Delta_{3,I}(x)]. \end{array}$$

Then, taking into consideration the forms of functions $\Delta_{3,R}(x)$ (57) and $\Delta_{3,I}(x)$ (58) and the following functions from Equations (74) and (75):

(85) $${\hat{\Delta }}_{R}(0,x)=Y_{0}(\alpha x)J_{0}(\beta x)-J_{0}(\alpha x)Y_{0}(\beta x),$$

(86) $${\hat{\Delta }}_{I}(0,x)=\varepsilon_{2}e^{-0.5\gamma^{*}}{[Y}_{0}(\alpha x)J_{1}(\beta x)-J_{0}(\alpha x)Y_{1}(\beta x)],$$

it was established that

(87) $${\hat{\Delta }}_{R}(0,x)+\varepsilon_{3}\Delta_{3,R}(x)=\Delta_{R}(x),\;{\hat{\Delta }}_{I}(0,x)-\varepsilon_{3}\Delta_{3,I}(x)=-\Delta_{I}(x),$$

where the functions $\Delta_{R}(x)$ and $\Delta_{I}(x)$ have the Formulas (54) and (55), respectively. Considering the results (87) in the right side of Equation (84), as well as the function Ψ(x) (52), the Equation (83) can be written in the form:

(88) $${\Theta^{\prime }}^{*}(0^{+},\tau )-K_{3}^{*}{\Theta^{\prime }}^{*}(0^{-},\tau )=-\frac{2}{\pi }\sqrt{\tau }\;\int_{0}^{\infty }F(x\sqrt{\tau })dx,\;\tau \geq 0.$$

Bearing in mind that [35]

(89) $$\int_{0}^{\infty }F(x)dx=\frac{\pi }{2},$$

from the Equation (88), it follows that the boundary condition (17) has been fulfilled.

Substituting ζ=1 ($\zeta_{1}=\beta ,\;\;\zeta_{2}=0$) in Equations (78) and (79) gives:

(90) $$e^{\gamma^{*}}{\Theta^{\prime }}^{*}(1^{+},\tau )=-\frac{2}{\pi }\sqrt{\tau }\;\int_{0}^{\infty }\frac{{\hat{\Phi }}_{1}(1,x)}{\Psi (x)}\;F(x\sqrt{\tau })dx,\;\tau \geq 0,$$

(91) $$K_{2}^{*}{\Theta^{\prime }}^{*}(1^{-},\tau )=-\frac{2\gamma^{*}K_{2}^{*}}{\pi^{2}}\sqrt{\tau }\;\int_{0}^{\infty }\frac{\Phi_{2}^{\prime }(1,x)}{x^{2}\Psi (x)}\;F(x\sqrt{\tau })dx,\;\tau \geq 0,$$

where, based on relations (77) and (81), it was found:

(92) $${\hat{\Phi }}_{1}(1^{+},x)=\Delta_{R}(x){\hat{\Delta }}_{R}(1,x)-\Delta_{I}(x){\hat{\Delta }}_{I}(1,x),\;K_{2}^{*}\Phi_{2}^{\prime }(1^{-},x)=-\varepsilon_{2}x\Delta_{I}(x).$$

From (74) and (75), it follows that [34]

(93) $${\hat{\Delta }}_{R}(1,x)=0,\;{\hat{\Delta }}_{I}(1,x)=\varepsilon_{2}e^{-0.5\gamma^{*}}{[J}_{1}(\beta x)Y_{0}(\beta x)-Y_{1}(\beta x)J_{0}(\beta x)]\equiv \varepsilon_{2}\gamma^{*}{\left(\pi x\right)}^{-1},$$

and the first Equation (92) takes the form

(94) $${\hat{\Phi }}_{1}(1^{+},x)=-\varepsilon_{2}\gamma^{*}{\left(\pi x\right)}^{-1}\Delta_{I}(x).$$

Considering Equations (93) and (94) in the right sides of Equations (90) and (91), it was determined:

(95) $$e^{\gamma^{*}}{\Theta^{\prime }}^{*}(1^{+},\tau )=\frac{2}{\pi^{2}}\varepsilon_{2}\gamma^{*}\sqrt{\tau }\;\int_{0}^{\infty }\frac{\Delta_{I}(x)}{x\Psi (x)}\;F(x\sqrt{\tau })dx=K_{2}^{*}{\Theta^{\prime }}^{*}(1^{-},\tau ),$$

which confirms that the obtained solutions meet the boundary condition (19).

Fulfillment of condition (20) of the disappearance of dimensionless temperature rises (60) and (61) for ζ→∞ was considered by rejecting the terms $e^{\zeta_{2}\sqrt{p}}$ and $e^{\zeta_{3}\sqrt{p}}$ while solving the boundary problem (23)–(33) and was verified by numerical calculations. Finally, it should be noted that solutions (59)–(61) also satisfy the initial condition (21).

## 5. Some Special Cases of Solution

In addition to the exact (in quadrature) solutions (59)–(62), appropriate asymptotic solutions were also obtained for small and large values of the Fourier number (dimensionless time) τ (11).

*Small values of* τ (*large values of parameter* p). Including Formulas (34)–(37) asymptotes of the modified Bessel functions for large argument values [34]:

(96) $$I_{n}(x)≅\frac{e^{x}}{\sqrt{2\pi x}},\;K_{n}(x)≅\sqrt{\frac{\pi }{2x}}\;e^{-x},\;n=0,\;1,\;\ldots ,$$

transformed solutions (31)–(33) were written in the forms:

(97) $${\bar{\Theta }}^{*}(\zeta ,p)≅\frac{e^{-0.25\gamma^{*}\zeta }}{\left(1+\varepsilon_{3}\right)}\frac{e^{-(\alpha -\zeta_{1})\sqrt{p}}}{p\sqrt{p}},\;0\leq \zeta <1,$$

(98) $${\bar{\Theta }}^{*}(\zeta ,p)≅\frac{2e^{-0.25\gamma^{*}}}{\left(1+\varepsilon_{2}e^{-0.5\gamma^{*}})(1+\varepsilon_{3}\right)}\frac{e^{-(\alpha -\beta +\zeta_{2})\sqrt{p}}}{p\sqrt{p}},\;\zeta \geq 1,$$

(99) $${\bar{\Theta }}^{*}(\zeta ,p)≅\frac{e^{-\zeta_{3}\sqrt{p}}}{(1+\varepsilon_{3})p\sqrt{p}},\;\zeta \leq 0,$$

where, based on definition (38), it was obtained:

(100) $$\alpha -\zeta_{1}=\frac{2}{\gamma^{*}}(1-e^{-0.5\gamma^{*}\zeta })\geq 0,\;\alpha -\beta +\zeta_{2}=\frac{2}{\gamma^{*}}(1-e^{-0.5\gamma^{*}})+\frac{\zeta -1}{\sqrt{k_{2}^{*}}}>0,\;\zeta_{3}\geq 0.$$

Proceeding from transforms (97)–(100) to the originals by means of the relation [36]:

(101) $$L^{-1}\left[\frac{e^{-\lambda \sqrt{p}}}{p\sqrt{p}};\tau \right]=2\sqrt{\tau \;}\mathrm{ierfc}\left(\frac{\lambda }{2\sqrt{\tau }}\right),\;\lambda \geq 0,$$

asymptotes of the dimensionless temperature rise in the initial moments of the heating process were found in the forms:

(102) $$\Theta^{*}(\zeta ,\tau )≅\frac{2e^{-0.25\gamma^{*}\zeta }\sqrt{\tau }}{(1+\varepsilon_{3})}\mathrm{ierfc}\left(\frac{\alpha -\zeta_{1}}{2\sqrt{\tau }}\right),\;0\leq \zeta <1,\;0\leq \tau <<1,$$

(103) $$\Theta^{*}(\zeta ,\tau )≅\frac{4e^{-0.25\gamma^{*}}\sqrt{\tau }}{\left(1+\varepsilon_{2}e^{-0.5\gamma^{*}})(1+\varepsilon_{3}\right)}\;\mathrm{ierfc}\left(\frac{\alpha -\beta +\zeta_{2}}{2\sqrt{\tau }}\right),\;\zeta \geq 1,\;0\leq \tau <<1,$$

(104) $$\Theta^{*}(\zeta ,\tau )≅\frac{2\sqrt{\tau }}{(1+\varepsilon_{3})}\mathrm{ierfc}\left(\frac{\zeta_{3}}{2\sqrt{\tau }}\right),\;\zeta \leq 0,\;0\leq \tau <<1,$$

where $\mathrm{ierfc}(x)=\pi^{-0.5}e^{-x^{2}}-x\;\mathrm{erfc}(x)$, erfc(x)=1−erf(x), and erf(x) are Gauss error functions [34].

*Large values of* τ (*small values of parameter* p). For small argument values, the modified Bessel functions behave as follow [34]:

(105) $$I_{0}(x)≅1,\;I_{1}(x)≅0.5x,\;K_{0}(x)≅-\ln(x),\;K_{1}(x)≅x^{-1}.$$

Considering asymptotes (105) in Equations (34)–(37), Laplace transforms (31)–(33) were presented in the forms:

(106) $${\bar{\Theta }}^{*}(\zeta ,p)≅\frac{1}{b}\left[\frac{1}{p\sqrt{p}(\sqrt{p}+c)}+\frac{\varsigma }{p(\sqrt{p}+c)}\right],\;0\leq \zeta \leq 1,$$

(107) $${\bar{\Theta }}^{*}(\zeta ,p)≅\frac{e^{-\zeta_{2}\sqrt{p}}}{b\;p\sqrt{p}(\sqrt{p}+c)},\;\;\zeta \geq 1,$$

(108) $${\bar{\Theta }}^{*}(\zeta ,p)≅\frac{e^{-\zeta_{3}\sqrt{p}}}{b}\left[\frac{1}{p\sqrt{p}(\sqrt{p}+c)}+\frac{a}{p(\sqrt{p}+c)}\right],\;\zeta \geq 0,$$

where

(109) $$a=\varepsilon_{2}\frac{\left(1-e^{-\gamma^{*}}\right)}{\gamma^{*}},\;b=1+\varepsilon_{3}a,\;c=\frac{\varepsilon_{2}+\varepsilon_{3}}{b},\;\varsigma =\varepsilon_{2}\frac{\left(e^{-\gamma^{*}\zeta }-e^{-\gamma^{*}}\right)}{\gamma^{*}}.$$

Bearing in mind that [36]

(110) $$L^{-1}\left[\frac{c\;e^{-\lambda \sqrt{p}}}{p(\sqrt{p}+c)};\tau \right]=\mathrm{erfc}\left(\frac{\lambda }{2\sqrt{\tau }}\right)-e^{\lambda c+c^{2}\tau }\mathrm{erfc}\left(\frac{\lambda }{2\sqrt{\tau }}+c\sqrt{\tau }\right),\;c>0,\;\lambda \geq 0,$$

(111) $$L^{-1}\left[\frac{c^{2}e^{-\lambda \sqrt{p}}}{p\sqrt{p}(\sqrt{p}+c)};\tau \right]=2c\sqrt{\frac{\tau }{\pi }}\;e^{-{\left(\frac{\lambda }{2\sqrt{\tau }}\right)}^{2}}-(1+\lambda c)\mathrm{erfc}\left(\frac{\lambda }{2\sqrt{\tau }}\right)+e^{\lambda c+c^{2}\tau }\mathrm{erfc}\left(\frac{\lambda }{2\sqrt{\tau }}+c\sqrt{\tau }\right),$$

the following asymptotes of the dimensionless temperature rise at high values of the Fourier number τ were obtained:

(112) $$\Theta^{*}(\zeta ,\tau )≅\frac{1}{\left(\varepsilon_{2}+\varepsilon_{3}\right)}\left\{2\sqrt{\frac{\tau }{\pi }}-\left(\frac{1}{c}-\varsigma \right)[1-e^{c^{2}\tau }\mathrm{erfc}(c\sqrt{\tau })]\right\},\;0\leq \zeta \leq 1,\tau >>1,$$

(113) $$\Theta^{*}(\zeta ,\tau )≅\frac{1}{\left(\varepsilon_{2}+\varepsilon_{3}\right)}\left\{2\sqrt{\frac{\tau }{\pi }}\;e^{-{\left(\frac{\zeta_{2}}{2\sqrt{\tau }}\right)}^{2}}-\left(\frac{1}{c}+\zeta_{2}\right)\mathrm{erfc}\left(\frac{\zeta_{2}}{2\sqrt{\tau }}\right)+\frac{1}{c}e^{c\zeta_{2}+c^{2}\tau }\mathrm{erfc}\left(\frac{\zeta_{2}}{2\sqrt{\tau }}+c\sqrt{\tau }\right)\right\},\;\;\zeta \geq 1,\;\tau >>1,$$

(114) $$\begin{array}{l} \Theta^{*}(\zeta ,\tau )≅\frac{1}{\left(\varepsilon_{2}+\varepsilon_{3}\right)}\left\{2\sqrt{\frac{\tau }{\pi }}\;e^{-{\left(\frac{\zeta_{3}}{2\sqrt{\tau }}\right)}^{2}}-\left(\frac{1}{c}-a+\zeta_{3}\right)\mathrm{erfc}\left(\frac{\zeta_{3}}{2\sqrt{\tau }}\right)\right.+\\ \;\;\;\;\;\;\;\;\;\;\;\;\;\;\;\;\;\;\;\;\;\;\;\;\;\;\;\;\;\;\;\;\;\;\;\;\;\;\left.\left(\frac{1}{c}-a\right)e^{c\zeta_{3}+c^{2}\tau }\mathrm{erfc}\left(\frac{\zeta_{3}}{2\sqrt{\tau }}+c\sqrt{\tau }\right)\right\},\;\;\zeta \leq 0,\;\tau >>1. \end{array}$$

On the friction surface ζ=0 and from Equations (38) and (109), it follows that $\zeta_{3}=0$ and ς=a, and from solutions (112) and (114) it was determined:

(115) $$\Theta^{*}(0^{+},\tau )=\Theta^{*}(0^{-},\tau )≅\frac{1}{\left(\varepsilon_{2}+\varepsilon_{3}\right)}\left\{2\sqrt{\frac{\tau }{\pi }}-\left(\frac{1}{c}-a\right)[1-e^{c^{2}\tau }\mathrm{erfc}(c\sqrt{\tau })]\right\},\;\tau >>1,$$

In a similar way, considering that on the interface ζ=1 we have $\zeta_{2}=0$ and ς=0, from solutions (112) and (113) it was found:

(116) $$\Theta^{*}(1^{+},\tau )=\Theta^{*}(1^{-},\tau )≅\frac{1}{\left(\varepsilon_{2}+\varepsilon_{3}\right)}\left\{2\sqrt{\frac{\tau }{\pi }}-\frac{1}{c}[1-e^{c^{2}\tau }\mathrm{erfc}(c\sqrt{\tau })]\right\},\;\tau >>1.$$

*Linearly descending temporal profile of specific friction power*. The exact solutions (59)–(61) presented above were obtained with the specific friction power $q_{0}$ remaining constant over time. Whereas, for modeling the frictional heating process in disc brake systems, the time profile of the specific friction power is most often used in the form [37]:

(117) $$q(t)=q_{0}q^{*}(t),\;q^{*}(t)=1-t\;t_{s}^{-1},\;0\leq t\leq t_{s},\;\tau >>1,$$

where $t_{s}$ is the moment of stopping the vehicle, and thus the final moment of the heating process. The dimensionless temperature rise ${\hat{\Theta }}^{*}(\zeta ,\tau )$, corresponding to the specific friction power (117) was found based on the Duhamel’s theorem [38,39]:

(118) $${\hat{\Theta }}^{*}(\zeta ,\tau )=\frac{\partial }{\partial \tau }\int_{0}^{\tau }q^{*}(\tau -s)\Theta^{*}(\zeta ,s)ds,\;\zeta \geq 0,\;0\leq \tau \leq \tau_{s},$$

where $\Theta^{*}(\zeta ,\tau )$ is dimensionless temperature increase (59)–(61), and function $q^{*}(\tau )$ has the following form:

(119) $$q^{*}(\tau )=1-\tau \;\tau_{s}^{-1},\;0\leq \tau \leq \tau_{s},\;\tau_{s}=k_{1}t_{s}d^{-2}.$$

Substituting solutions (59)–(61) and function $q^{*}(\tau )$ (119) as the integrand in the right side of Equation (118), after performing the integration according to the methodology from [29], it was found:

(120) $$\Theta^{*}(\zeta ,\tau )=2\sqrt{\frac{\tau }{\pi }}\left[\frac{P(\tau )}{\varepsilon_{2}+\varepsilon_{3}}+\frac{2}{\pi }e^{-0.5\gamma^{*}\zeta }\int_{0}^{\infty }\frac{\Phi_{1}(\zeta ,x)}{x\Psi (x)}Q(\tau ,x)dx\right],\;0\leq \zeta \leq 1,\;0\leq \tau \leq \tau_{s},$$

(121) $$\Theta^{*}(\zeta ,\tau )=2\sqrt{\frac{\tau }{\pi }}\left[\frac{P(\tau )}{\varepsilon_{2}+\varepsilon_{3}}-\frac{2\gamma^{*}}{\pi^{2}}\int_{0}^{\infty }\frac{\Phi_{2}(\zeta ,x)}{x^{2}\Psi (x)}Q(\tau ,x)dx\right],\;\zeta \geq 1,\;0\leq \tau \leq \tau_{s},$$

(122) $$\Theta^{*}(\zeta ,\tau )=2\sqrt{\frac{\tau }{\pi }}\left[\frac{P(\tau )}{\varepsilon_{2}+\varepsilon_{3}}+\frac{2}{\pi }\int_{0}^{\infty }\frac{\Phi_{3}(\zeta ,x)}{x\Psi (x)}Q(\tau ,x)dx\right],\;\zeta \leq 0,\;0\leq \tau \leq \tau_{s},$$

where

(123) $$P(\tau )=1-\frac{2\tau }{3\tau_{s}},\;Q(\tau ,x)=\left(1+\frac{1}{x^{2}\tau_{s}}\right)F(x\sqrt{\tau })-\frac{2}{\sqrt{\pi }x^{2}\tau_{s}},$$

and functions $\Phi_{k}(\zeta ,x)$, k=1,2,3 are determined from (49)–(51) and (53)–(58).

## 6. Numerical Analysis

Calculations were carried out for a friction pair consisting of two half-spaces; one has a two-component FGM coating applied on the substrate, and the other (counterbody) slides on the working surface of the FGC with constant or linearly decreasing velocity. The base and core of the FGM are, respectively, zirconium dioxide ZrO_2_ and titanium alloy Ti-6Al-4V. The substrate is ChNMKh gray cast iron, and the counterbody is cermet FMC-11. The properties of these materials, at initial temperature $T_{0}=20{}^{\circ}C$, are given in Table 1.

The specific heat and density of the coating material were determined according to the mixture law, based on the data for ZrO_2_ and Ti-6Al-4V (Table 1). For equal participation of the volume fractions of the base and core components, it was established that $c_{1}=495.55\;J\;{\mathrm{kg}}^{-1}K^{-1}$ and $\rho_{1}=\;5266.98\mathrm{kg}\;m^{-3}$. The dimensionless gradient parameter of the FGM and the time of braking were equal to $\gamma^{*}=\ln(K_{1,2}\;K_{1,1}^{-1})=1.26$ and $\tau_{s}=1$, respectively.

**Figure 2:**
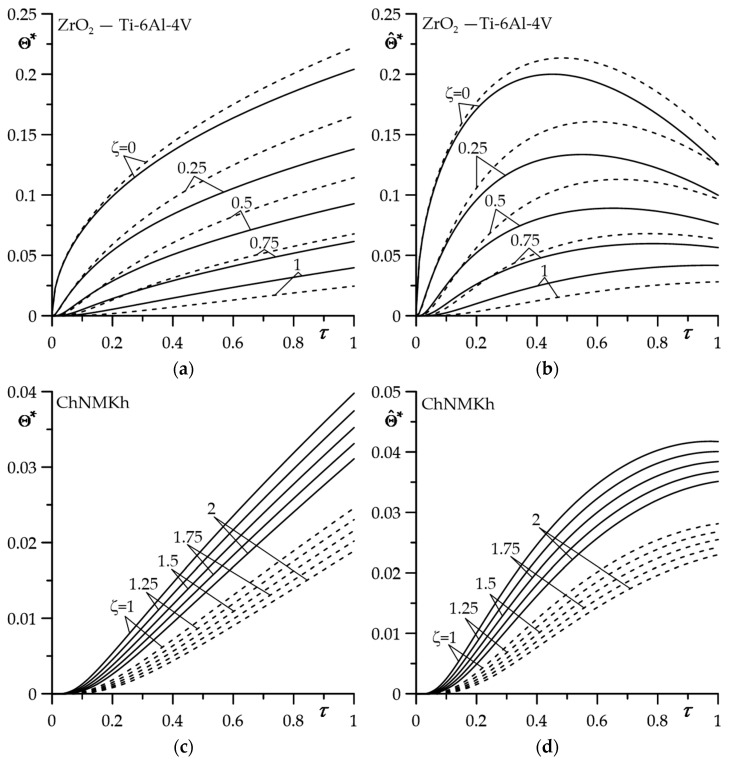

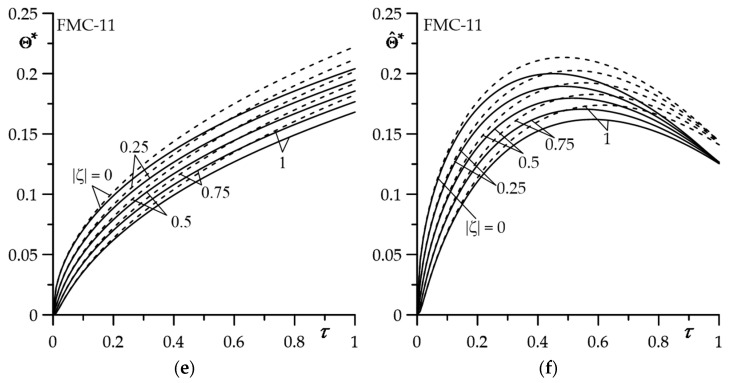
Evolutions of dimensionless temperature rises $\Theta^{*}(\zeta ,\tau )$ (59)–(63) (**a**,**c**,**e**) and ${\hat{\Theta }}^{*}(\zeta ,\tau )$ (120)–(123) (**b**,**d**,**f**) for selected values of dimensionless distance from the contact surface ζ in: (**a**,**b**) The coating; (**c**,**d**) the substrate; (**e**,**f**) the counterbody. Solid lines—FGC, dashed lines—coating made of ZrO_2_.

**Figure 3:**
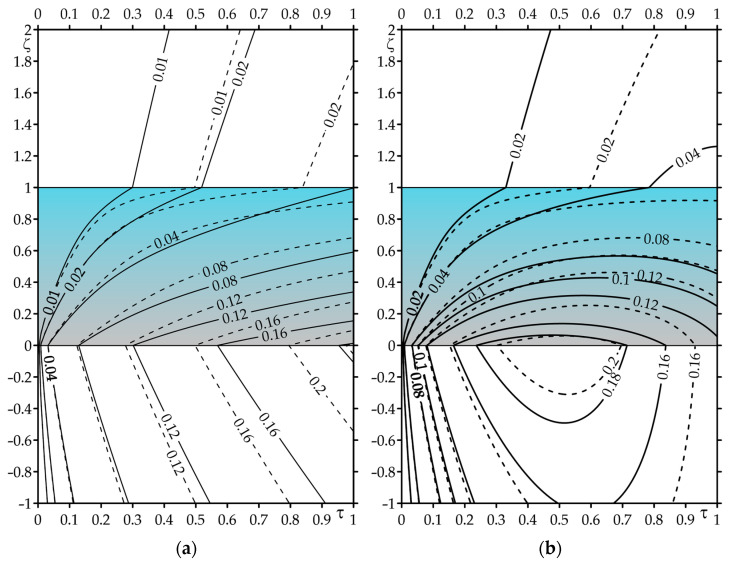
Isotherms: (**a**) $\Theta^{*}(\zeta ,\tau )$ (59)–(63); (**b**) ${\hat{\Theta }}^{*}(\zeta ,\tau )$ (120)–(123). Solid lines—FGC, dashed lines—coating made of ZrO_2_.

The results of the calculations for dimensionless temperature rises $\Theta^{*}(\zeta ,\tau )$ (59)–(63) for constant, and ${\hat{\Theta }}^{*}(\zeta ,\tau )$ (120)–(123) for linearly decreasing specific power of friction are presented in Figure 2 (evolutions) and Figure 3 (isotherms). For numerical integration, the QAGI procedure from the QUADPACK library was implemented [40]. A numerical analysis was carried out to compare the results obtained for the applied FGC on the substrate (solid lines) with the corresponding data found for a homogeneous coating made entirely of zirconium dioxide (dashed lines).

The Ti-6Al-4V titanium alloy, with its thermal conductivity 3.5 times greater than zirconium dioxide ZrO_2_, effectively dissipates heat from the contact surface. As a result, the temperature of the FGC is lower compared to that determined using a homogeneous material (Figure 2a,d). Such a temperature mode changes to the opposite, starting from the interface (ζ=1) and further into the substrate (ζ>1) (Figure 2b,e). The ChNMKh cast iron used for the disc substrate has significantly (about 7.8 times) higher thermal conductivity than the Ti-6Al-4V titanium alloy. As a result, the temperature of the substrate at a fixed distance from the interface during the entire heat generation process is lower in the case of a homogeneous coating made of zirconium dioxide. In both cases, the level of substrate temperature is much (by an order of magnitude) lower than the coating temperature level. The change in the counterbody temperature over time (FMC-11, Figure 2c,f) is quantitatively and qualitatively similar to the evolution of the coating temperature, shown in Figure 2a,d. However, there are some features of the temporal profiles of the counterbody temperature that differ from the corresponding time courses of the coating temperature. Firstly, the effect of the FGM on the temperature of the counterbody is much lower than in the coating itself. Finally, the drop in the temperature in the counterbody (pad) with the distance from the contact surface is much slower than in the coating.

The spatial-temporal distributions of the dimensionless temperature rises for the constant and time-dependent intensity of specific friction power are demonstrated in Figure 3. They confirm the result from Figure 2 that the effective absorber of heat generated by friction on the contact surface is the coating made of the considered two-component FGM. It is clearly visible here that it plays the role of a thermal barrier, effectively protecting the substrate against overheating.

**Figure 4:**
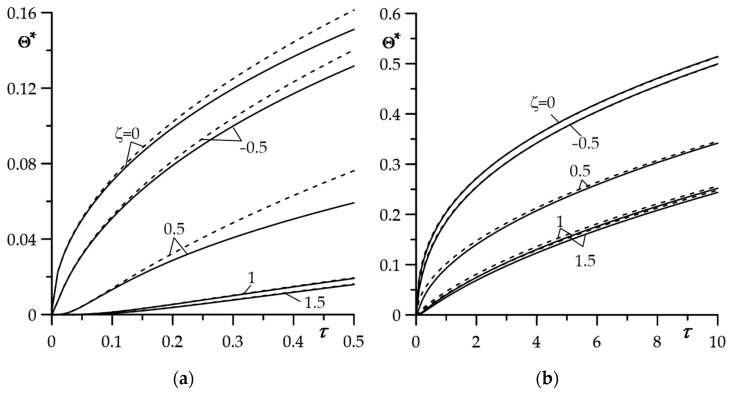
Evolution of the dimensionless temperature rises $\Theta^{*}(\zeta ,\tau )$ obtained using the exact (59)–(63) (solid lines) and asymptotic (dashed lines) solutions: (**a**) For small (102)–(104); (**b**) for large (112)–(114) Fourier numbers τ for selected values of dimensionless spatial variable ζ.

The asymptotic solutions for small (102)–(104) and large (112)–(114) values of the Fourier number τ (11) were an effective tool for estimating the temperature of the considered system in the case of constant specific friction power. The calculation results presented in Figure 4 show that the satisfactory convergence of the exact and asymptotic (at small τ) solutions takes place in the range 0≤τ≤0.5 (Figure 4a). However, it is surprising that the results obtained using the exact and asymptotic (for large τ) solutions show terrific agreement in almost the entire range of Fourier number changes (Figure 4b). It is all the more valuable due to the fact that asymptotic solutions, unlike exact solutions, do not require numerical integration.

**Figure 5:**
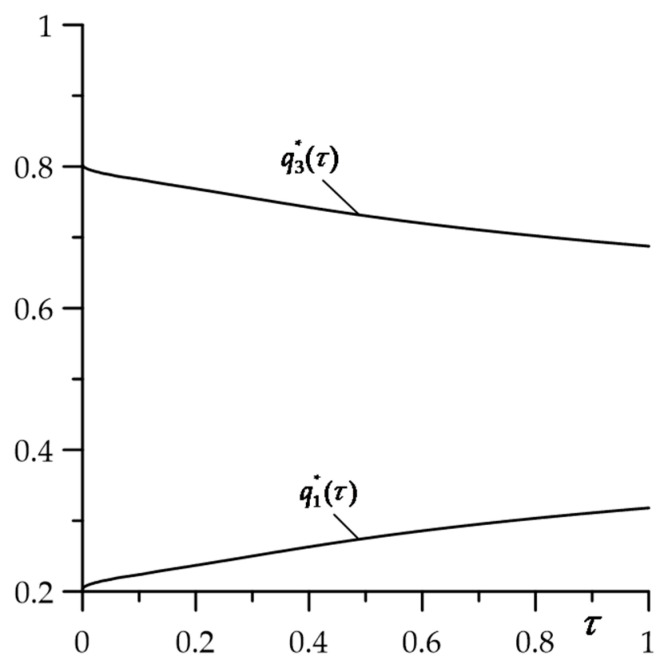
Evolutions of the dimensionless intensities of heat fluxes $q_{l}^{*}(\tau )$, *l* = 1, 3, defined by Equation (124).

Based on the Fourier law, dimensionless intensities of heat fluxes for constant specific power of friction, directed perpendicularly to the contact surface ζ=0 to the insides of the FGC and homogeneous counterbody were written in the forms, respectively:

(124) $$q_{1}^{*}(\tau )={\Theta^{\prime }}^{*}(0^{+},\tau ),\;q_{3}^{*}(\tau )=-K_{3}^{*}{\Theta^{\prime }}^{*}(0^{-},\tau ),\;\tau \geq 0,$$

where derivatives were determined from Formulas (78) and (80). Calculations carried out on the basis of Equation (124) demonstrated that the greater part of the heat generated on the contact area was absorbed by the cermet pad, which had much better heat conduction capabilities compared to the zirconium dioxide (Figure 5). At the initial moments of the heating process, about 80% of the heat is absorbed by the pad, and only 20% by the FGC. With the elapse of heating time, the amount of heat directed to the pad (coating) decreases (increases) and, for τ=1, it is equal to 70% (30%).

## 7. Conclusions

An analytical model to determining the temperature field of a three-element friction system is developed, consisting of a substrate with protective coating deposited on the surface and a counterbody. The materials of the substrate and counterbody are homogeneous, while the coating is made of a functionally gradient material with an exponentially increasing thermal conductivity coefficient along the thickness. The counterbody slides on the surface of the coating, as a result of which heat is generated, and the elements of the system heat up. This type of system is used to simulate the frictional heating process in the pair, i.e., the pad (counterbody) and the coated disc (substrate with a coating). The crucial element of the model is the boundary value problem of heat conduction, considering the generation of heat due to friction at a constant and linearly decreasing specific friction power. Exact and asymptotic solutions of such a problem were obtained. Based on the achieved solutions, a numerical analysis was carried out for a cermet (FMK-11) brake pad sliding on the surface of the FGC (ZrO_2_–Ti-6Al-4V) perfectly connected to a cast iron (ChNMKh) disc. It is concluded that:

1. Application of the selected functionally graded coating on the friction surfaces of the disc is an effective tool to lower the maximum temperature of the system.
2. The temporal profile of the specific friction power has a significant influence on the spatial-temporal distribution of the isotherms in the coating and the pad. The temperature of the disc is by an order of magnitude lower than the temperatures of the coating and the pad.
3. Asymptotic solutions for small and large values of the Fourier number can be used for quick estimation of temperature with high accuracy for all elements of the system.
4. The higher part of the heat (≈3/4) generated on the contact surface due to friction is absorbed by the pad, and the smaller part (≈1/4) is absorbed by the FGC.

It should be noted that protective coatings made of functionally graded materials are applied in braking systems for the following reasons: On the one hand, to reduce wear on the friction surface of the disc it is desirable to use a component with high wear resistance in the FGC (in the analyzed case—ZrO2). On the other hand, these materials usually have a low capacity to dissipate the heat generated by friction on the contact surface. The problem of lowering the possibly high temperature on the friction surface is solved by simultaneously using in the FGC a component with a much higher thermal conductivity coefficient than the previous one (in our case, Ti-6Al-4V). The numerical analysis carried out on the basis of the proposed analytical model showed that the last task of the FGC of the selected type is completely solved.

In the future, authors intend to expand research on the heating process in brakes with a functionally graded friction element and to develop models to investigate the influence of imperfect thermal contact of friction, convective cooling with the environment, as well as the finite thickness of friction pair elements.

## Figures and Tables

**Table 1:** Materials properties [23,29].

Material	Thermal Conductivity ${\mathrm{Wm}}^{-1}K^{-1}$	Specific Heat $J\;{\mathrm{kg}}^{-1}K^{-1}$	Density $\;\mathrm{kg}\;m^{-3}$
ZrO_2_	$K_{1,1}=1.94$	$c_{1,1}=\;452.83$	$\rho_{1,1}=\;6102.16$
Ti-6Al-4V	$K_{1,2}=6.87$	$c_{1,2}=\;538.08$	$\rho_{1,2}=\;4431.79$
ChNMKh	$K_{2}=52.17$	$c_{2}=\;444.6$	$\rho_{2}=\;7100$
FMC-11	$K_{3}=35.0$	$c_{3}=\;478.9$	$\rho_{3}=4700$

## Data Availability

No new data were created or analyzed in this study. Data sharing is not applicable to this article.

## Nomenclature

c	Specific heat ($J\;{\mathrm{kg}}^{-1}K^{-1}$)
d	Thickness of coating (m)
f	Coefficient of friction (dimensionless)
Imp	Imaginary part of a complex Laplace parameter
$I_{n}(\cdot )$	Modified Bessel functions of the *n*th order of the first kind
$J_{n}(\cdot )$	Bessel functions of the *n*th order of the first kind
$K_{n}(\cdot )$	Modified Bessel functions of the *n*th order of the second kind
k	Thermal diffusivity ($m^{2}s^{-1}$)
K	Thermal conductivity ($W\;m^{-1}K^{-1}$)
p	Parameter of the Laplace integral transform (dimensionless)
$p_{0}$	Nominal pressure on the contact surface (Pa)
Rep	Real part of a complex Laplace parameter
q	Specific power of friction ($W\;m^{-2}$)
$q_{0}$	Nominal value of the specific friction power ($W\;m^{-2}$)
t	Time (s)
$t_{s}$	Stop moment of the process (s)
T	Temperature (°C)
$T_{0}$	Initial temperature (°C)
$V_{0}$	Sliding velocity ($m\;s^{-1}$)
$Y_{n}(\cdot )$	Bessel functions of the *n*th order of the second kind
x	Spatial coordinate in tangential direction (m)
y	Spatial coordinate in radial direction (m)
z	Spatial coordinate in axial direction (m)
$\gamma^{*}$	Gradient parameter of FGM
Λ	Temperature rise scaling factor (°C)
ε	Dimensionless coefficient of thermal activity
Θ	Temperature rise (°C)
$\Theta^{*}$	Dimensionless temperature rise
ρ	Density ($\mathrm{kg}\;m^{-3}$)
τ	Dimensionless time
$\tau_{s}$	Dimensionless stop time
ζ	Dimensionless spatial coordinate in axial direction
subscript *l*	Number of the element of the system
